# Thymodepressin—Unforeseen Immunosuppressor

**DOI:** 10.3390/molecules26216550

**Published:** 2021-10-29

**Authors:** Vladislav I. Deigin, Julia E. Vinogradova, Dmitry L Vinogradov, Marina S. Krasilshchikova, Vadim T. Ivanov

**Affiliations:** 1 Institute of Bioorganic Chemistry, Russian Academy of Sciences, Miklukho-Maklaya st., 16/10, 117997 Moscow, Russia; msk@ibch.ru (M.S.K.); ivavt@ibch.ru (V.T.I.); 2 Hematology Department, Sechenov First Moscow State Medical University, Russian MOH, Moscow 8-2 Trubetskaya str., 119991 Moscow, Russia; jvinogr@gmail.com (J.E.V.); wind007@mail.ru (D.L.V.)

**Keywords:** new peptide immunosuppressor, reciprocal activity peptide enantiomers, autoimmune treatment, comparison with cyclosporine A

## Abstract

The paper summarizes the available information concerning the biological properties and biomedical applications of Thymodepressin. This synthetic peptide drug displays pronounced immunoinhibitory activity across a wide range of conditions in vitro and in vivo. The history of its unforeseen discovery is briefly reviewed, and the current as well as potential expansion areas of medicinal practice are outlined. Additional experimental evidence is obtained, demonstrating several potential advantages of Thymodepressin over another actively used immunosuppressor drug, cyclosporin A.

## 1. Introduction

### 1.1. Historical Background

Since the 1960s, the central organs of the animal immune system (bone marrow, thymus, spleen) have attracted the attention of researchers as a source of biologically active compounds that affect the function of the immune system and are promising for use in practical medicine. In the mid-1960s, A. Goldstein showed that one of the fractions of the acetone extract of calf thymus (the so-called thymosin fraction 5 with a molecular weight of 1000–15,000 Da) has a pronounced biological activity, restoring the suppressed immune response in mice [1,2]. Similar drugs, thymomodulin [3,4], thymulin [5], and Tactivin [6], differing in the details of the technological process for the extraction of the thymus of cattle, found a certain application as a first generation of broad bioactivity spectrum immunostimulants in medical practice. Subsequent study of thymosin fraction 5 led to the discovery of the first peptide hormones that regulate the functioning of the mammalian immune system, namely thymosin α1 (28 amino acid residues) [7,8]), thymosin β4 (43 residues), and thymopoietin (49 residues) [9]. It was soon shown that the bovine spleen extract contains a peptide called splenin, which differs from thymopoietin by a single Asp → Glu substitution at position 34 of the peptide chain. At the same time, it was found that their shortened synthetic analogs, thymopentin and splenopentin, which correspond to sites 32–36 of the original hormones and, therefore, form their active functional centers, have activity close to natural hormones [10,11,12]. Several examples of the use of thymopentin and splenopentin as pharmacological agents have been described [13]. In addition, for some time, thymopentin was actively used in the early stages of the selection of AIDS therapy [14]. Thymus hormones also include the nine-membered peptide thymulin (serum thymic factor), isolated in 1977 from pig blood, then sequenced [15] and after that found in the thymosin fraction 5 [16,17]. The functional form of thymulin is its complex with zinc. Thymulin induces T-cell differentiation and enhances several functions of T-cell subpopulations in health and immunodeficiency [18].

Research into peptide regulators produced by another central organ of the immune system, the bone marrow, has developed in a similar pattern since the 1970s. The peptide complex named myelopid was developed and approved in Russia for various immunodeficiency states from the supernatant of the bone marrow cell culture of pigs and calves. The drug restored the indices of the B- and T-systems of immunity, stimulating both the production of antibodies and the functional activity of immunocompetent cells [19,20,21,22]. Later, six individual peptides (MP1-MP6) were isolated from the complex preparation, synthesized, and tested for activity in experimental models. It was found that some peptides (hexapeptide MP-1) inhibited the activity of T-suppressor lymphocytes and stimulated the production of the interleukin 2 by T lymphocytes (MP-2) [23,24].

In 1982 in the Soviet Union (and in the 1990s in Russia), another extract of cattle thymus predominantly containing low molecular weight (below 10 kDa) peptides was approved in Russia as a drug, Thymalin, for treating various immunodeficiency states and diseases [25,26,27].

The search for active components of the extract led to the dipeptide GluTrp, which displayed a broad range of immunoprotective effects and was registered in 1990 as a drug Thymogen [28,29,30]. It was found that Thymogen enhances the expression of Thy-1 receptors on lymphocytes, accelerates the differentiation processes of various subpopulations of lymphocytes and regulates the number and the ratio of T-helpers and T-suppressors. Furthermore, the drug stimulates the activity of cAMP-dependent protein kinases in the lymphocytes of the thymus and the spleen. A change in differentiation receptors accompanied incubation of T-lymphocyte precursors with the dipeptide: the expression of the SC-1 antigen was replaced by the expression of the Thy-1 antigen, which indicates the transformation of the T-lymphocyte precursor into a mature T-cell. Being a popular immunomodulatory drug, Thymogen shows no allergic or other side reactions. Statistics indicate that over 25 million of patients have used it during the last 30 years.

The Thymogen molecular mechanisms of action and other immunoactive peptides mentioned above have not yet been established and are the subject of active research. If for thymopoietin, splenin, and thymulin protein receptors of cell membranes are considered as targets [11,12,31,32,33] in the case of Thymogen, it has been suggested that this dipeptide (like many other short peptides) targets the promoter regions of the DNA double helix in lymphocyte cells. It is assumed that such binding transforms the “silent” heterochromatin into active euchromatin, which leads to an increase in the availability of respective genes for transcription [34,35].

### 1.2. Discovery of Thymodepressin

For Thymogen, a detailed study of the dependence of its activity on the chemical structure [29,30,35,36,37] was carried out. Dozens of peptide analogs have been synthesized and tested for bioactivity. The primary test was study of the ability of the peptide to restore the regeneration of surface E-thymocyte receptors after their treatment with trypsin. It was shown that the type of bond between Glu and Trp residues (via α- or γ-carboxyl) does not affect the level of immunostimulatory activity. At the same time, reversal of configuration, as expected, dramatically changed the level of activity: substitutions l-Glu → d-Glu and l-Trp → d-Trp, regardless of the type of bond (α or γ), led to its significant (though not complete) drop [29,37]. The reversal of the configuration of both asymmetric (chiral) centers led to an unexpected result: both DD isomers, d-Glu-d-Trp (α-bond) and d-Glu(d-Trp) (γ-bond), not only did not show immunostimulating activity (which was expected) but proved to be effective inhibitors of regeneration under the same conditions in which the LL-isomers had a stimulating effect. We have not found examples of such opposite actions of enantiomers in the literature. As a rule, enantiomers are either inactive in biological tests (which is understandable given the chirality of the targets) or exhibit a type of activity unrelated to the initial one (a classic example is the notorious thalidomide [38]). The only exception is ionophore antibiotics (enniatins A and B [39], valinomycin [40]) when the key functional step is binding with an achiral metal ion.

**Figure 1:**
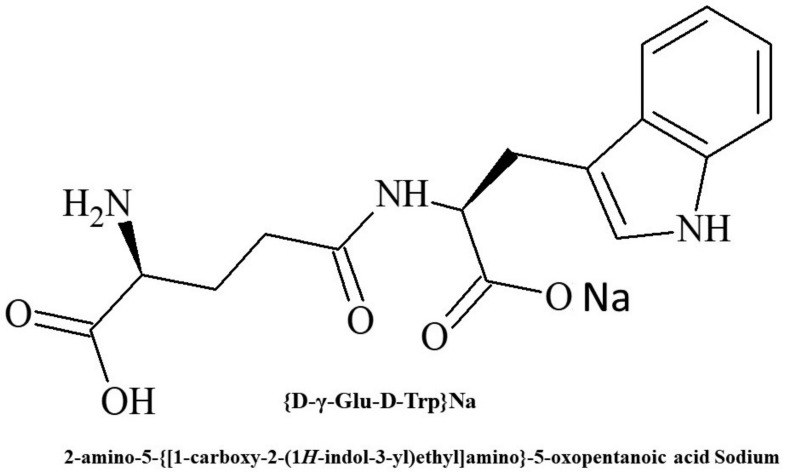
Chemical structure of Thymodepressin.

The reasons for the unique activity reversal found for Thymogen require further investigation. Nevertheless, according to a number of indicators, the effects of DD isomers turned out to be attractive for use in medicine and based on one of them (the γ-DD isomer), the drug Thymodepressin (Figure 1) was developed and registered in 2008 in Russia [41,42,43]. 

At that time, the all-D structure of peptide pharmaceutical was a unique case. In 2016 etelcalcetide, an octapeptide containing 7 d-amino acid residues appeared on the market as a drug for treating secondary hyperparathyroidism [44]. This peptide inhibits the cationic calcium-binding receptors by an allosteric mechanism involving reversible disulfide formation, with apparently no analogy to Thymodepressin.

The main biological effects induced by Thymodepressin will be discussed below. Its advantages and disadvantages as an immunosuppressant will be compared with the properties of another currently used drug cyclosporine A. The prospect of creating new generation drugs based on Thymodepressin will be considered.

### 1.3. Thymodepressin as an Immunosuppressor. Biological Activity and Medical Applications

A systemic study of Thymodepressin in cell cultures and animal models [45,46,47] led to the conclusion that bone marrow hematopoietic progenitors (CD34+ cells) are the primary target of its action. Thymodepressin, both in vitro and in vivo, affects the initial stages of hemopoiesis, reducing the number of committed (CFU-C-8, CFU-GM) cells and the percentage of cells in the S-phase of the cell cycle. As a result, administration of the peptide leads to a dose-dependent transient decrease in the number of leucocytes in the blood of the experimental animal. The primary method used to prove this mechanism of action was the thymidine suicide method [48,49], which allows one to correctly measure the percentage of dividing cells (in the S phase of the cell cycle). Its main principle is the incubation of a bone marrow suspension with 3H-thymidine, which kills cells in the S-phase, after which the difference between the number of spleen colonies from untreated and treated bone marrow can be used to judge the proliferation rate at this level. The experiments were carried out in a wide range of conditions with various combinations of doses of Thymodepressin, in combination with cytostatic and without it, with and without allogeneic bone marrow [35,36,50].

A number of related effects have been observed:

- Thymodepressin inhibits the proliferation of PHA-stimulated human lymphocytes, as well as the spontaneous proliferation of human thymus cells [51].
- Thymodepressin inhibits migration of CD34+ cells from the bone marrow into peripheral blood both in normal and tumor-bearing animals [50].
- Prophylactic application of Thymodepressin leads to earlier restoration of the population of both committed (CFU-C-8) and pluripotent (CFU-C-12) hematopoietic precursors after exposure to the cytostatic cytosine arabinoside (cytosar) [52].
- Thymodepressin injected into donor mice two days before irradiation at a dose of 4 Gy promotes more intensive restoration of the population of hematopoietic progenitor cells than the control group [53].
- Thymodepressin suppresses the development of graft-versus-host-reaction after allogenic bone marrow transplantation in mice [42].

The immuno-inhibiting properties of Thymodepressin turned out to be in demand for use in medicine, and, as already mentioned, it was registered in Russia as a drug. Currently, Thymodepressin is most actively used for the treatment of autoimmune and allergic processes caused by lymphocyte-mediated hyperimmune reactions. Such diseases are now observed in 8% of the population of industrialized countries. These include psoriasis, atopic dermatitis, lichen planus, autoimmune cytopenia (autoimmune thrombocytopenia, autoimmune hemolytic anemia, two- and three-cell cytopenia), and many other syndromes [45,54,55,56,57].

There is no doubt that the use of Thymodepressin is promising in confronting cancer due to its ability to reduce the harmful effects of radiation therapy and cytostatic (in the latter case, by reducing the output of hematopoietic stem cells into differentiation with the simultaneous use of Thymodepressin and cytostatic, without canceling the antitumor effect).

Another level of action of Thymodepressin on hematopoiesis is the suppression of CD25+ and CD69+ markers of activation of mature T-lymphocytes, promoting a regenerating effect on the epidermis, reducing epidermal hyperplasia in the affected skin areas during skin tumor processes (fungal mycosis, Sesari’s syndrome, autoimmune dermatitis).

In addition, Thymodepressin suppresses the migration of CD34+ cells from the bone marrow of patients into the bloodstream reducing the likelihood of metastases and the frequency of recurrent tumors. However, in general, the use of Thymodepressin in anticancer therapy is still at an early stage [41].

The advantages of Thymodepressin as a drug include its wide therapeutic range and the absence of toxic side reactions. In contrast to most immunosuppressors, Thymodepressin displays a regulatory rather than cytotoxic effect, and its inhibitory action is reversible. In addition, Thymodepressin acts only on activated cell clones, without suppressing memory cell’s activity responsible for the acquired immunity. Thymodepressin is administered by subcutaneous or intramuscular injection, as well as by intranasal application. Despite resistance to standard proteases (due to the d-configuration of its constituent amino acids), an attempt to apply Thymodepressin orally did not lead to success due to considerable rise of the effective dose [58].

A significant area of the practical application of immunosuppressants is transplantation, where drugs of this class are the principal means for combating the rejection of transplanted tissues and organs. The most used drugs for this purpose are the cyclic peptide cyclosporin A (registered as Sandimmun Neoral) [59] and the macrolide antibiotic Tacrolimus [60]. These products of fungal metabolism form stable complexes with the protein cyclophilin (proline cis-trans isomerase), which block T-cell activation and signaling by calcineurin phosphatase interaction. Following the experimental assessment of Thymodepressin potential to prevent graft rejection [42], we conducted in this work a head-to-head comparison between Thymodepressin and cyclosporin A in several tests. First, the suppressive activity of both drugs was compared in a standard model of the induction of an autoimmune response in mice [57]. After that an initial comparison of their effect was carried out on graft-versus-host-reaction developed after allogenic spleen cell transplantation in mice.

## 2. Results and Discussion

### 2.1. Comparison of Thymodepressin and Cyclosporin A in an Experimental Autoimmunity Model

**Figure 2:**
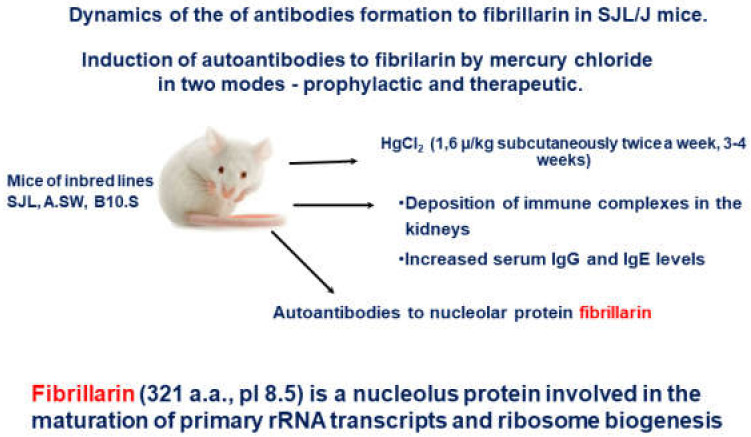
Experimental design of the of antibodies formation to fibrillarin in SJL/J mice.

The SJL/J mice model of mercury-induced autoimmunity [61,62] was used to examine the immunosuppressive effects of the two above mentioned peptides. In this strain of mice, the production of anti-fibrillarin antibodies (AFA) was induced with the administrations of low doses of mercury chloride (HgCl_2_). The SJL/J mice regularly administrated by sub-lethal doses of HgCl_2_ develop a systemic autoimmune response. They generate antinuclear autoantibodies, polyclonal B-cell activation, and systemic immune-complex deposits and are often used as a favorable autoimmune model. Besides the AFA production, autoimmune processes are initiated by such treatment which models several aspects of human systemic autoimmune diseases [63] (Figure 2).

**Figure 3:**
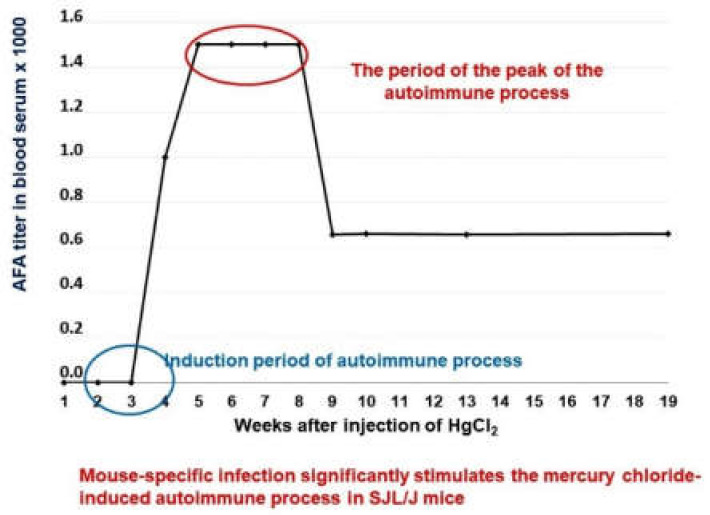
Dynamics of the of antibodies formation to fibrillarin in SJL/J mice.

The level of anti-fibrillarin antibodies was measured as an indicator of the autoimmune response (Figure 3). Using this model, we have compared the suppressive effects of Thymodepressin and cyclosporine A (Sandimmun, Novartis Pharma Stein AG, Switzerland) in two regiments of treatment, prophylactic (preventive) and therapeutic, that is, before the induction of autoimmunity and after its full development. Cyclosporin A is widely used to prevent organ rejection in clinical transplantation and to treat autoimmune diseases. The effects of cyclosporine A on mercury induced autoimmunity are described in [64].

#### 2.1.1. Development of Anti-Fibrillarin Antibodies (AFA) in Control Groups

All mice in the control group developed AFA after four weeks of HgCl_2_ treatment in a medium titer (1:800). IIF assessed the titer of serum AFA after the treatment with HgCl_2_.

#### 2.1.2. The Effect of Cyclosporin A in the Prophylactic Regimen

**Figure 4:**
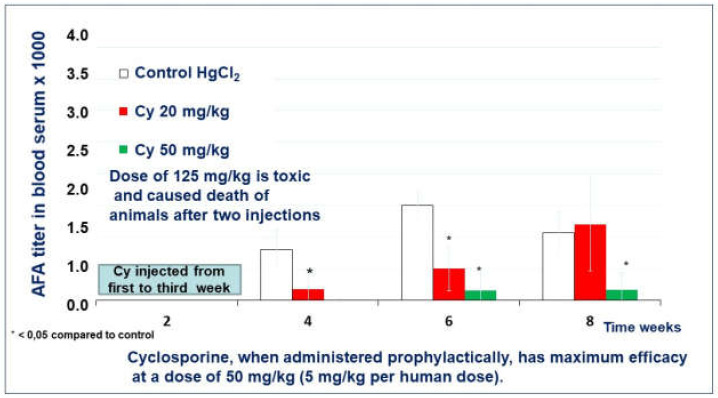
The effect of cyclosporin A in a prophylactic regimen.

After four weeks of the experiment, the mice who received cyclosporine A in a low dose (20 mg/kg) in the preventive regimen developed AFA in a low titer (1:180). (Figure 4). The mice of the control group developed AFA in significantly higher titers, 1:800 (Control). In the 6th week of the experiment, the titer of AFA in all groups grew twice. In the 8th week, the mean titer of AFA in cyclosporine A treated mice reached the AFA level in the control group (1:2000 and 1:1071, respectively).

A single mouse of the group treated with a medium dose of cyclosporine A (50 mg/kg) developed AFA after four weeks of the experiment (Figure 4). However, no mice except this one developed AFA during all eight weeks of observation. For example, in the 8th week of the experiment, the mean titer of AFA in the cyclosporine A treated group was only 1:160.

A high dose (125 mg/kg) of the cyclosporine A was toxic for SJL/J mice and caused 60% death in the group after the third injection.

#### 2.1.3. The Effect of Thymodepressin in the Prophylactic Regimen

**Figure 5:**
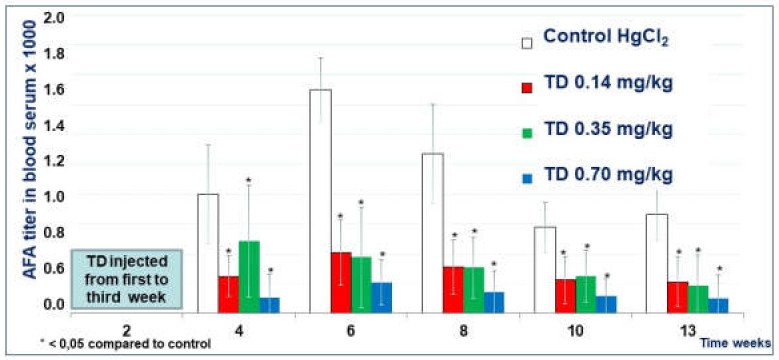
The effect of Thymodepressin in a prophylactic regimen.

The titer of serum AFA in mice treated with all three doses of Thymodepressin in the prophylactic regimen was significantly lower than in the Control group (Figure 5) during all times of observation. The experiment’s most significant suppression by the 0.7 mg/kg, observed in week 6, was the classical peak of autoantibody production in mice’s mercury-induced autoimmune model. The mean titer of AFA in mice that received high dose Thymodepressin did not statistically differ from mice treated with low dose Thymodepressin (Figure 5).

#### 2.1.4. Comparison of the Effects of Thymodepressin and Cyclosporin A in the Prophylactic Regimen

**Figure 6:**
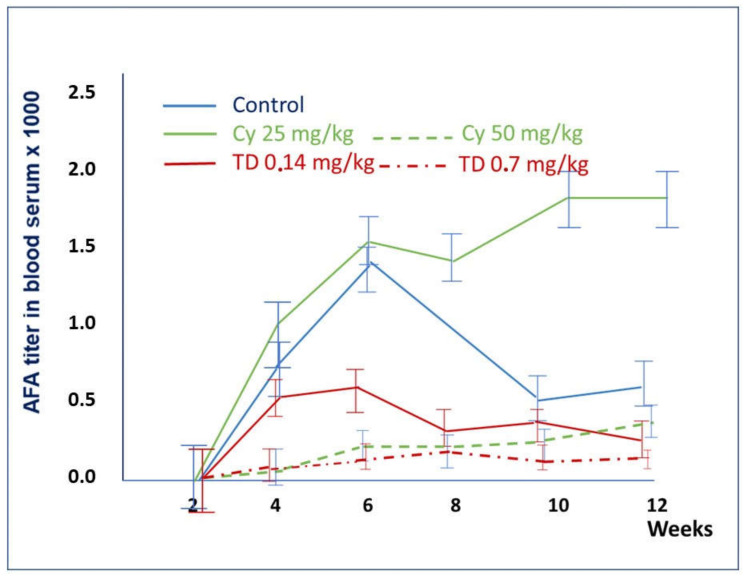
A comparison of the effects of Thymodepressin and Cyclosporin A in the prophylactic regimen. The titers of serum AFA after the immunosuppressive treatment with Thymodepressin or cyclosporin A in different doses in the prophylactic regimen. Values are mean. Vertical bars denote 0.95 confidence intervals. Significant differences were analyzed using ANOVA followed by Kruskal-Wallis Test, Van der Waerden Two-Sample Test, Van der Waerden One-Way Analysis). Significant differences (*p* < 0.05) were found between the groups: Thymodepressin and Control on 4th, 6th, 8th, 10th, and 12th weeks of the experiment. The cyclosporine A (50 mg/kg) and Control group’s difference was on weeks 4th 6th and 10th of the experiment.

The results described above are summarized in Table 1 and Figure 6. Low doses of Thymodepressin and cyclosporine A (0.14 mg/kg and 20 mg/kg, respectively) on the sixth week showed similar effects on mice’s autoimmune process. However, the suppression of the autoimmune process was more pronounced. The titer of AFA differs significantly on weeks six and eight of the experiment when in cyclosporin A treated mice, it rose to 1: 1200, whereas in Thymodepressin treated mice, the titer was 1: 313.

On the fourth and sixth weeks of the experiment, the mean titer of AFA in mice treated with Thymodepressin in low dose (0.14 mg/kg) was comparable with dose (50 mg/kg) in mice treated with cyclosporine A. However, after week 8th of the experiment, the mean titer of AFA in Thymodepressin (0.70 mg/kg) treated mice declined and reached the level of cyclosporin A (50 mg/kg) treated group.

As mentioned above, high doses of cyclosporin A were toxic for three out of five mice.

In the prophylactic regimen, mice received both peptides for three weeks before the first injection of HgCl_2_. The titer of anti-fibrillarin antibodies was assessed in mice three weeks after injection either with HgCl_2_ only (group 1), with Thymodepressin (groups 2–4), or cyclosporin A (groups 5, 6) at 4th-8th weeks. The mean value and standard deviation are indicated (Table 1).

#### 2.1.5. Effect of Cyclosporin A in the Therapeutic Regimen

**Figure 7:**
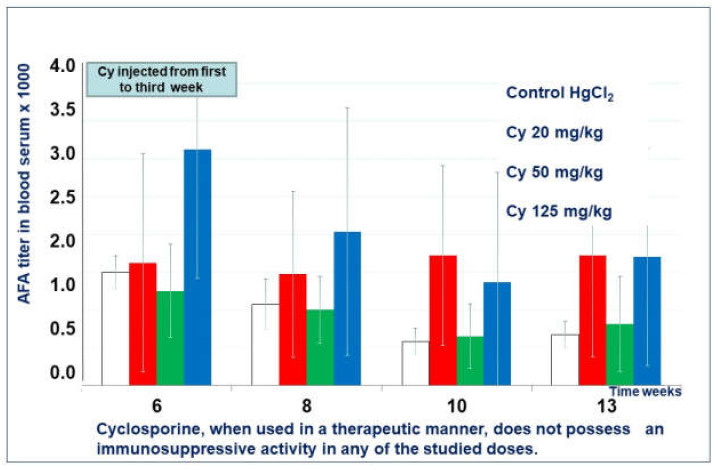
Cyclosporine A effect in therapeutic regimen.

Mice treated with cyclosporin A in all doses in the therapeutic regimen did not show any more significant differences in the mean titer of AFA than the control group (Figure 7).

#### 2.1.6. Effect of Thymodepressin in the Therapeutic Regimen

**Figure 8:**
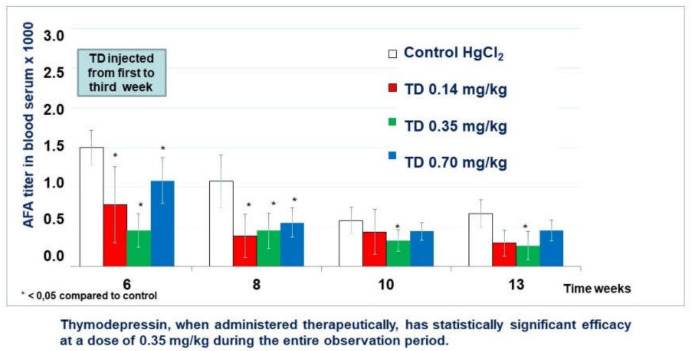
Thymodepressin (TD) effect in therapeutic regimen.

Thymodepressin in low dose markedly decreased the titer of AFA as compared with the control group. Differences became statistically significant in weeks 6, 8, and 12 (Figure 8).

Thymodepressin in medium dose significantly inhibited AFA development’s autoimmunity process from the first week after Thymodepressin treatment and through observation periods (weeks 6th–12th).

Thymodepressin in high dose affected the suppression of AFA titer at the same level as Thymodepressin in medium dose. Statistically significant titer was lower than in the control group (weeks 6th–12th). 

#### 2.1.7. Comparison of the Effects of Thymodepressin and Cyclosporin A in a Therapeutic Regimen

**Figure 9:**
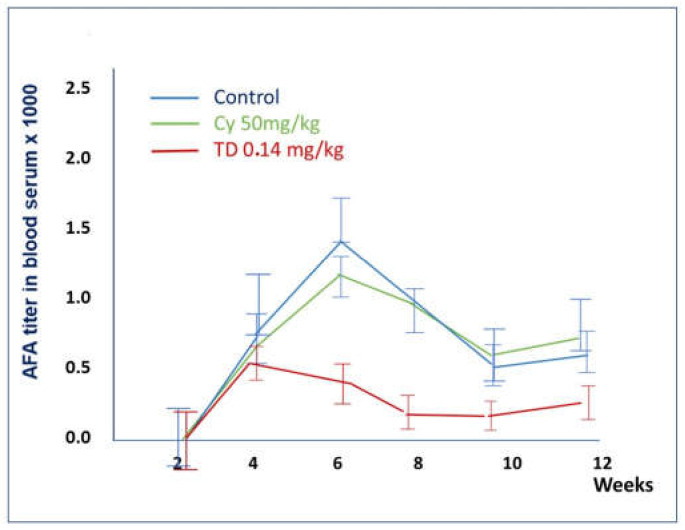
Comparison of the effects of Thymodepressin and cyclosporine A in the therapeutic regimen.

The results described above are summarized in Table 2 and Figure 9.

Thymodepressin markedly changed the autoimmune process’s character for AFA compared to cyclosporin A in all tested doses.

TD in low dose statistically significantly reduced the AFA titer, compared to Cyclosporine A, throughout the entire observation period (Figure 9).

TD in medium dose significantly reduced the titer of AFA at all weeks. Cyclosporin A in this group did not change the titer of AFA comparing to the control (Figure 9).

In the therapeutic regimen, mice received an immunosuppressive agent for three weeks after five weeks of HgCl_2_ injections at the maximum of the autoimmune response. The titer of anti-fibrillarin antibodies was assessed in mice injected after five weeks treatment either with HgCl_2_ only (group 1), with Thymodepressin (groups 2–4), or cyclosporine A (groups 5–7) at weeks 6–12. The mean values and standard deviations are indicated.

#### 2.1.8. Summary

In a model of an autoimmune process induced by mercury chloride in SJL/J mice, cyclosporine A and Thymodepressin showed activity in a prophylactic regimen. Cyclosporin A was effective only over a relatively narrow dose range of 20–50 mg/kg. The 125 mg/kg dose was found to be toxic and lethal to mice after two injections. The most pronounced immunosuppressive effect was achieved when using cyclosporine A at a dose of 50 mg/kg. This dose is comparable to the optimal dose for treating human autoimmune diseases (5 mg/kg/day).

Cyclosporine A did not show immunosuppressive efficacy at any dose when administered in a therapeutic regimen. It is known that the use of cyclosporin-containing drugs requires constant monitoring of the concentration of cyclosporine A in the blood plasma, and excess of its therapeutic concentration can lead to complications [65].

When used prophylactically, Thymodepressin had a pronounced immunosuppressive effect at all studied doses: 0.14, 0.35, and 0.7 mg/kg (Figure 5). Moreover, this effect of the drug persisted for a long time, namely 10 weeks after the end of the drug administration.

With therapeutic use, the most pronounced immunosuppressive effect was manifested at 0.35 mg/kg (Figure 8).

In the experiment, Thymodepressin had a distinct immunosuppressive effect at doses ranging from 0.14 mg/kg to 0.7 mg/kg. For comparison, the most effective dose of cyclosporine A was significantly higher and amounted to about 50% of the LD_50_ (50 mg/kg). The maximum Thymodepressin dose used in our study (0.70 mg/kg) showing statistically significant immunosuppressive activity is 70 times lower than the respective dose of cyclosporin A (50 mg/kg). Bearing in mind the toxicity of cyclosporin A [66], these results provide another argument favoring further attempts to apply Thymodepressin. Moreover, in contrast to cyclosporine A, Thymodepressin is highly soluble in aqueous media and does not require solvents other than 0.9% NaCl.

In conclusion, both preparations suppress the autoantibodies in this experimental model in the prophylactic regimen. However, cyclosporine A did not show immunosuppressive efficacy at any dose when administered in a therapeutic regimen. On the contrary, Thymodepressin shows distinct immunosuppressive effects both in prophylactic and therapeutic modes. In other words, cyclosporine A inhibits the autoimmune response in a notably narrower range of autoimmunity development stages than Thymodepressin. The mechanistic background of that phenomenon requires further study.

### 2.2. Comparison of the Effects of Thymodepressin and Cyclosporine A on the Graft-Versus-Host-Reaction Developed after Allogenic Spleen Cells Transplantation

In this experiment, we used the graft versus host (GVHD) model for the experiments based on the induction of chronic GVHD in intact hybrid animals described in [67]. Mice (CBA × C57Bl/6)F1 were used as recipients, and C57Bl/6 mice were used as spleen cell donors. Animals were injected twice with a 7-day interval of 10^5^ allogeneic parent spleen cells. GVHD development has been observed for 21 days. Thymodepressin was i/p administrated at an optimal dose of 10 μg/kg, experimentally obtained in the in vivo dose-response experiments [37]. The drug started to be administered 24 h after the first injection of spleen cells in five-day courses for three weeks. Cyclosporine A was applied in standard dose of 2.0 mg/0.1 mL/mouse subcutaneously, with the same regimen as Tymodepressin.

The splenomegaly lesions were measured after 21 days of disease and used as a degree of the chronic GVHD. The effect of the preparations was calculated as the percentage of splenomegaly suppression related to the control (Equation (1) The splenomegaly lesions were measured in% to control).

(1) $$\frac{\left(\mathrm{SI}\mathrm{control}-\mathrm{SI}\mathrm{intact}\right)-\left(\mathrm{SI}\mathrm{preparation}-\mathrm{SI}\mathrm{intact}\right)}{\mathrm{SI}\mathrm{control}-\mathrm{SI}\mathrm{intact}}\times 100\%$$

The percentage of splenomegaly suppression (GVHD) after the treatment was calculated according to [65]. The presented results indicate (Table 3) that Tymodepressin significantly suppresses the GVHD development in mice (70.7%). On the other hand, the cyclosporin A activity was 34.6%. For both peptides, the activity has not reached the intact control.

The experiment described above is clearly an initial attempt to estimate the prospect of Thymodepressin application for preventing graft-versus-host-reaction. The result obtained clearly support further attempts to apply Thymodepressin in transplantation practices.

## 3. Conclusions and Prospects

The discovery of Thymodepressin led to the emergence of a new type of bioactive peptides, significantly different in terms of the novelty of their properties from all known physiologically active substances, both natural and synthetic. Thymodepressin has already found several essential areas of application in medical practice and continues to expand the range of indications for further use. Although its use as a drug in several important areas (cancer, organ, and tissue transplantation) is at an early stage, it seems advisable to study Thymodepressin itself further and to create new analogs with improved properties with the spectrum of activity modified in the desired direction. An example of such a development of events is the design and synthesis, based on the original platform [68] of a cyclic analog of Thymodepressin, exhibiting total activity when administered orally [37,58]. There is no doubt that further progress in Thymodepressin application, similarly to other immunoactive drugs, will strongly depend on the level of understanding of their molecular mechanisms of action. We believe that intensive search in that direction should remain a priority in the near future.

## 4. Materials and Methods

### 4.1. Reagents

- mercury chloride (HgCl_2_) (Sigma, Burlington, MA, USA);
- sodium chloride (NaCl) (JSC “Biochemist”, Moscow, Russia);
- Sandimmun^®^ (Novartis Pharma AG, Basel, Switzerland);
- Thymodepressin^®^ (Peptos Pharma, Moscow, Russia);
- ethanol 96%;
- Phosphate-buffered saline PBS (140 mM NaCl, 2.7 mM KCl, 10 mM Na_2_HPO_4_, 1.8 mM KH_2_PO_4_, pH 7.2–7.4);
- HEp-2 cell preparations (Bio-Rad Laboratories, Hercules, CA, USA);
- Moviol (Sigma, Ronkonkoma, NY, USA);
- DABCO (1,4-Diazobicyclo [2.2.2] octane) (Sigma, Ronkonkoma, NY, USA);
- Antibodies:
  - rabbit polyclonal antibodies to fibrillarin (Abcam, Boston, MA, USA);
  - goat antibodies to mouse immunoglobulins conjugated to fluorescein isothiocyanate (IMTEK, Moscow, Russia);
  - goat antibodies to rabbit immunoglobulins conjugated with tetrarodamine isothiocyanate (SouthernBiotech Associates, Birmingham, AL, USA);
  - goat antibodies to human immunoglobulins conjugated with tetrarodamine isothiocyanate (Santa Cruz Biotechnology, Dallas, TX, USA);
  - antibodies to mouse immunoglobulins conjugated with horseradish peroxidase (Sigma, Ronkonkoma, NY, USA).

### 4.2. Equipment

- thermostat “Biological Thermostat BT 120” (Labsystems, East Yorkshire, UK);
- centrifuge “Eppendorf 5804 R” (Eppendorf, Hamburg, Germany);
- MiniSpin Plus centrifuge (Eppendorf, Hamburg, Germany);
- Beckman J2-21 centrifuge (Beckman, Indianapolis, IN, USA);
- fluorescent microscope Axiovert 200 (Carl Zeiss, Göttingen, Germany);
- 13-bit monochrome digital camera CoolSnapcf (Roper Scientific, Sarasota, FL, USA);
- Axioscop A1 microscope (Carl Zeiss, Germany);
- digital color camera AxioCam MRc5 (Carl Zeiss, Germany);
- cryotome (MICRO, Walldorf, Germany);
- pH meter MP220 (Mettler Toledo, Columbus, OH, USA);
- electrophoretic chamber Mighty Small II (AmershamPharmaciaBiotech, Piscataway, NJ, USA);
- chamber for semi-dry electroblotting (BioRad, Hercules, CA, USA); PowerPac HC power supply (BioRad);
- EPS 601 power supply (AmershamParmaciaBiotech, USA);
- Direct-Q 5 tap water purification system (Millipore, Burlington, MA, USA);
- automatic pipettes of various calibrations (Eppendorf, Framingham, MA, USA);
- plastic tubes of 1.5 mL (Eppendorf, Germany).

### 4.3. Methods

#### 4.3.1. Detection of Autoantibodies to Fibrillarin

##### Indirect Immunofluorescence Method

The detection of autoantibodies to fibrillarin in blood serum was performed by indirect immunocytochemistry on monolayer human HEp-2 cells and NIH/3T3 mouse cells. NIH/3T3 cells were washed from the culture medium in 0.1 M PBS and fixed with 4% paraformaldehyde (PFA) in 0.1 M PBS for 15 min at room temperature. WNext, washed with 0.1 M PBS three times for 10 min and incubated with 0.1% Triton X-100 in PBS for 10 min on ice, washed with 0.1 M PBS four times for 5 min. Then, the cells were incubated with animal sera taken in various dilutions for 45 min at 37 ° C in a humid chamber. The cells were washed in 0.1 M PBS three times for 10 min and incubated with secondary antibodies for 45 min at 37 °C in a humid chamber. Antibodies to mouse IgG conjugated with fluorescein isothiocyanate (FITC) at a dilution of 1: 200 were used as secondary antibodies. To reduce nonspecific binding, 0.05% Tween-20 was added to antibodies. The cells were washed in 0.1 M PBS three times for 10 min and stained with DAPI (4,6-diamidino-2-phenylindole) chromatin dye at a final concentration of 1 μg/mL for 10 min at room temperature. The cells were embedded in Moviol containing DABCO (1,4-diazobicyclo[2.2.2]octane).

The cells were examined under an Axiovert 200 microscope (Carl Zeiss, Germany) using a 40× PlanNeoFluar objective (NA 0.75) and photographed using a CoolSnapcf monochrome 13-bit camera (Roper Scientific, USA). The images were processed using Adobe Photoshop (version 9.0).

The titer of autoantibodies to fibrillarin was defined as the maximum dilution of sera at which they ceased to stain nucleoli, a traditional approach used in the analysis of autoimmune sera in the laboratory and clinical practice [69].

##### Western Blotting

The electrophoretic separation of proteins was carried out according to the standard method proposed by [70]. NIH/3T3 cells were lysed on ice in buffer A containing 50 mM Tris-(hydroxymethyl)-aminomethane (pH 7.5), 150 mM NaCl, 10% glycerol, 0.5% Triton X-100, and a cocktail of protease inhibitors (Sigma) using a pestle homogenizer. Before lysis, the number of cells in each sample was counted using a Goryaev chamber. The Lowry method (Peterson’s modification) used the Protein Assay Kit (Sigma) to determine the total concentration of proteins in the lysates was determined by. The Lowry method (Peterson’s modification) using the Protein Assay Kit (Sigma), following the manufacturer’s recommendations.

Before electrophoretic separation, 5-fold Laemmli’s buffer was added to the prepared lysates containing 250 mM Tris-(hydroxymethyl)-aminomethane (pH 6.8), 50% glycerol, 10% sodium dodecyl sulfate (SDS), 500 mM β-mercaptoethanol, and 0.5% bromophenol blue. The samples were heat-treated at 100 °C for 5 min. Lysates obtained from the same number of cells containing at least 50 μg of total protein were applied to each pocket of 12% PAGE. The electrophoretic separation of proteins was carried out at 60 V for concentrating proteins in the “top” (concentrating) gel and at 160 V for separation in an electrophoresis chamber. After protein separation, the gel was incubated in a buffer for semi-dry electroblotting containing 50 mM Tris-(hydroxymethyl)-aminomethane, 38 mM glycine, 0.1% SDS, and 20% methanol. Proteins were transferred to a nitrocellulose membrane (0.22 μm, Millipore, USA) at a constant voltage of 20 V for 30 min. The membrane was incubated with mouse sera (dilution 1:200), and then with antibodies to mouse immunoglobulins conjugated with horseradish peroxidase (dilution 1:2000) in TBST buffer containing 20 mM Tris-(hydroxymethyl)-aminomethane (pH 7.6), 150 mM NaCl, 0.05% Tween-20. According to the manufacturer’s instructions, the membrane was developed by chemiluminescence using the ECL + Plus kit (Amersham Pharmacia Biotech, UK). The Hyperfilm X-ray film developer (Amersham Pharmacia Biotech) contained 7% metol and 7% hydroquinone, and the fixer contained 25% Na_2_S_2_O_3_ and 2.5% Na_2_SO_3_.

##### Colocalization of an Autoantigen Recognized by the Sera of Autoimmune Mice with FiBrillarin

HEp-2 cells were incubated with primary antibodies for 45 min at 37 ° C in a humid chamber. A mixture of mice autoimmune sera (dilution 1:100) with commercial rabbit polyclonal antibodies to fibrillarin (dilution 1:200), or with the serum of a patient with rheumatoid arthritis (dilution 1:100), has recognized fibrillarin in human cells. After incubation with antibodies, the cells were washed in 0.1 M PBS three times for 10 min and incubated with secondary antibodies containing 0.05% Tween-20 for 45 min at 37 °C in a humid chamber. As secondary antibodies, we used a mixture of antibodies to mouse IgG conjugated to FITC (dilution 1:200), to antibodies to rabbit IgG conjugated to tetrarodamine isothiocyanate (TRITC) (dilution 1:100), or to antibodies to human IgG conjugated to TRITZ (dilution 1:100). The cells were washed with 0.1 M PBS three times for 10 min and stained with DAPI at a final concentration of 1 μg/mL for 10 min at room temperature. The cells were embedded in Moviol containing DABCO. The preparations were examined under an Axiovert 200 microscope as described in Section 1.1.

#### 4.3.2. Identification of Immune Complexes in the Kidneys

Deposits of immune complexes in the kidney were detected on frozen sections in the reaction of direct immunofluorescence. Organ sections with a thickness of 5 μm were obtained using a cryotome. Cryosections were placed in 0.1 M cold (4–10 °C) PBS for 1 min, and then fixed with absolute acetone for 5 min at room temperature and dried in air. This approach is standard when working with cryosections of organs from autoimmune animals [71]. Cryosections were incubated with antibodies to total mouse immunoglobulins (IgA, IgG, IgM) conjugated with FITC (dilution 1:200) for 45 min at 37 °C in a humid chamber, washed in PBS three times for 10 min, counterstained with DAFI dye for 10 min at room temperature and imprisoned in Moviol. Cryosections were examined under an Axiovert 200 microscope as described in Section 1.1.

### 4.4. Mice

Specific pathogen-free (SPF) SJL/J 6–8-week-old female mice were purchased from the Animal Breeding Facility-Branch of The Shemyakin-Ovchinnikov Institute of Bioorganic Chemistry, Pushchino (Moscow Region, Russia). Mice were kept in SPF conditions with the natural night and day light-cycle and received a standard diet and drinking water ad libitum. Eight groups of mice, each consisting of 5–8 mice with an approximate weight of 20 g, were used (Table 1).

### 4.5. Substances Used for Treatment

HgCl_2_ of the analytical grade was purchased from Sigma Aldrich and dissolved in sterile 0.9% NaCl to the final concentration of 0.4 mg/mL.

Commercial Thymodepressin is a sterile 1 mg/mL solution in 0.9% NaCl. It was diluted in sterile 0.9% NaCl to a required concentration immediately before injection. For example, mice treated with the maximum dose of Thymodepressin (0.7 mg /kg b.w.) received 0.5 mL of a solution containing 0.028 mg of Thymodepressin in 1 mL of 0.9% NaCl.

Cyclosporin A was used in the commercial preparation of Sandimmun I.V. (Novartis Pharma), one mL of which contains 50 mg of cyclosporin A, 650 mg of castor oil, and 278 mg of 96% ethanol. Sandimmun was diluted in sterile 0.9% NaCl to the required concentration of cyclosporine A immediately before injection. For example, mice treated with the maximum dose of cyclosporin A (125 mg/kg b.w.) received 0.5 mL of a solution containing 5 mg of cyclosporin A, 65 mg of castor oil, and 27.8 mg of 96% ethanol in 1 mL of 0.9% NaCl.

### 4.6. The Induction of an Autoimmune Process

All groups of mice were administered 0.1 mL of 0.4 mg/mL HgCl_2_ (1.6 mg/kg b.w.) subcutaneously twice per week during the experiment (8 weeks) to induce the production of autoantibodies.

All groups of mice were administered Thymodepressin or cyclosporin A intraperitoneally five times per week for three weeks beginning from the first HgCl_2_ injection.

### 4.7. Blood Sampling

Mice were bled by a retroorbital puncture every second week. The maximum volume of blood taken was 50 μL. The obtained sera were kept at −200 ° C until usage.

### 4.8. Autoantibody Detection

Indirect immunofluorescence was performed as previously described [69]. Diluted sera were applied to a monolayer of fixed Hep-2 cells (Bio-Rad Laboratories, Redmond, WA, USA) and recovered with FITC-conjugated goat anti-mouse IgG antibodies (Sigma, Ronkonkoma, NY, USA). Samples were examined under an epifluorescence microscope Axiovert 200 (Carl Zeiss, Germany). The highest dilution of sera at which the nucleolar fluorescence (Figure 2) could be detected was defined as the titer of anti-fibrillarin antibodies, AFA [71].

### 4.9. Statistical Methods

Statistical processing of experimental data was carried out using the ANOVA program, accompanied by Kruskal–Wallis, Van der Werden, and two-sample tests and Van der Werden one-way analysis. Data are presented as means and 0.95 confidence intervals or as means and standard deviations (when determining the number of follicles with germinal centers in the spleen). Changes in the studied parameters were considered significant at *p* < 0.05.

## Figures and Tables

**Table 1:** The titers of AFA after treatment by cyclosporine A or Thymodepressin in the preventive regimen.

Groups	Regimens of HgCl_2_ Administration	Time (in Weeks)		
		4th	6th	8th
1	HgCl_2_ only	800 ± 331	1500 ± 214	1071 ± 333
HgCl_2_ simultaneously with:				
2	Thymodepressin 0.14 mg/kg	250 ± 137	406 ± 219	313 ± 180
3	Thymodepressin 0.35 mg/kg	486 ± 349	379 ± 337	307 ± 201
4	Thymodepressin 0.7 mg/kg	108 ± 155	208 ± 153	142 ± 144
5	Cyclosporin A 20 mg/kg	180 ± 282	500 ± 346	1200 ± 733
6	Cyclosporin A 50 mg/kg	10 ± 20	150 ± 294	160 ± 290

**Table 2:** The titers of AFA after treatment by cyclosporin A or Thymodepressin in the therapeutic regimen.

Groups	Regimens of HgCl_2_ Administration	Time (in Weeks)			
		6th	8th	10th	13th
1	HgCl_2_ only	800 ± 331	1500 ± 214	1071 ± 333	667 ± 175
2	Thymodepressin 0.14 mg/kg	775 ± 481	383 ± 270	433 ± 284	294 ± 14
3	Thymodepressin 0.35 mg/kg	450 ± 213	450 ± 227	325 ± 137	258 ± 179
4	Thymodepressin 0.7 mg/kg	1079 ± 288	550 ± 185	443 ± 212	452 ± 135
5	Cyclosporin A 20 mg/kg	1620 ± 1446	1470 ± 1099	1720 ± 1188	1720 ± 1343
6	Cyclosporin A 50 mg/kg	1250 ± 620	1000 ± 438	650 ± 427	810 ± 635
7	Cyclosporin A 125 mg/kg	3125 ± 1715	2033 ± 1641	1367 ± 1455	1700 ± 1444

**Table 3:** Influence of immunosuppressors on splenomegaly development.

Group	Mice #	Splenomegaly Index	% Suppression
Control	15	10.2 ± 0.8 *	-
Cyclosporine A	15	8.8 ± 0.5	34.6
Thymodepressin	15	7.3 ± 1.0 *	70.7
Intact	10	6.1 ± 1.0	

* *p* < 0.05.

## Sample Availability

Samples of the thymodepressin are available from the authors.
